# Personal

**Published:** 1890-11

**Authors:** 


### Personal.

Dr. E. Storck has removed his office and residence to No. 220 East Eagle street, where he should be sought and addressed.

James A. Lydston, M. D., Ph. D., late chief of the Eye and Ear Department, Pension Bureau, Washington, D. C., has been elected to the chair of Chemistry in the College of Physicians and Surgeons, Chicago.

